# *N*-Docosahexanoylethanolamine Reduces Microglial Activation and Improves Hippocampal Plasticity in a Murine Model of Neuroinflammation

**DOI:** 10.3390/ijms21249703

**Published:** 2020-12-19

**Authors:** Anna Tyrtyshnaia, Anatoly Bondar, Sophia Konovalova, Ruslan Sultanov, Igor Manzhulo

**Affiliations:** A.V. Zhirmunsky National Scientific Center of Marine Biology, Far Eastern Branch, Russian Academy of Sciences, Palchevskogo Str, 17, 690041 Vladivostok, Russia; bondar.av@dvfu.ru (A.B.); sofanasrew@gmail.com (S.K.); sultanovruslan90@ya.ru (R.S.); i-manzhulo@bk.ru (I.M.)

**Keywords:** neuroinflammation, *N*-docosahexanoylethanolamine, DHEA, synaptamide, long-term potentiation, neurogenesis, microglia, cytokines, dendrites

## Abstract

Chronic neuroinflammation is a common pathogenetic link in the development of various neurological and neurodegenerative diseases. Thus, a detailed study of neuroinflammation and the development of drugs that reduce or eliminate the negative effect of neuroinflammation on cognitive processes are among the top priorities of modern neurobiology. *N*-docosahexanoylethanolamine (DHEA, synaptamide) is an endogenous metabolite and structural analog of anandamide, an essential endocannabinoid produced from arachidonic acid. Our study aims to elucidate the pharmacological activity of synaptamide in lipopolysaccharide (LPS)-induced neuroinflammation. Memory deficits in animals were determined using behavioral tests. To study the effects of LPS (750 µg/kg/day, 7 days) and synaptamide (10 mg/kg/day, 7 days) on synaptic plasticity, long-term potentiation was examined in the CA1 area of acute hippocampal slices. The Golgi–Cox method allowed us to assess neuronal morphology. The production of inflammatory factors and receptors was assessed using ELISA and immunohistochemistry. During the study, functional, structural, and plastic changes within the hippocampus were identified. We found a beneficial effect of synaptamide on hippocampal synaptic plasticity and morphological characteristics of neurons. Synaptamide treatment recovered hippocampal neurogenesis, suppressed microglial activation, and significantly improved hippocampus-dependent memory. The basis of the phenomena described above is probably the powerful anti-inflammatory activity of synaptamide, as shown in our study and several previous works.

## 1. Introduction

Modern society is characterized by a tendency towards a growth in life expectancy and an increase in the percentage of aged people. Aging contributes to the development of neurodegenerative pathologies, many of which are associated with dementia. According to the World Health Organization, there are approximately 50 million people with dementia worldwide. There are about 10 million new cases of the disease every year (WHO Newsletter, 21 September 2020, https://www.who.int/news-room/fact-sheets/detail/dementia). In this situation, it is very important to find new approaches for the treatment and prevention of neurodegenerative pathologies. This demand has led to an increase in the number of studies aimed at the treatment of cognitive disorders.

Numerous studies have found that neuroinflammation is a common component of various neurological and neurodegenerative diseases [1]. The presence of chronic neuroinflammation is characteristic of such diseases as Alzheimer’s [2], Parkinson’s [3], bipolar disorder [4], major depressive disorder [5], etc. Although a causal link between neuroinflammation and neurodegenerative disease development requires a more detailed investigation, neuroinflammation is considered to be a major neurodestructive factor ultimately leading to cognitive deficits. Presumably, neuroinflammation contributes to cognitive impairment in many neurological and neurodegenerative diseases. The role of neuroinflammation in the impairment of cognitive functions has been demonstrated recently. It was shown that the acute neuroinflammation induced by bacterial lipopolysaccharides results in working memory deterioration in aging mice [6,7]. Moreover, a deficiency in context-dependent recognition of objects has been shown in acute neuroinflammation [8]. A similar decrease in long-term memory associated with neuroinflammation was observed in the novel object recognition test with a retention period of 24 h [9]. Thus, experimental data suggest that neuroinflammation is one of the factors leading to cognitive impairments.

Among the possible mechanisms of neuroinflammation’s involvement in cognitive impairment, the most evident is microglial activation. Activation of microglia in neuroinflammation leads to oxidative and nitrosative stress development, increased release of reactive oxygen and nitrogen species, and consequently to the damaging of mitochondria [10]. Mitochondrial damage reduces energy for axonal and other intracellular transports, resulting in reduced synaptic plasticity. Another possible explanation for the cognitive decline during neuroinflammation may be the phenomenon of excitotoxicity, a pathological process leading to nerve cell damage and death by excessive NMDA- and AMPA-receptor activation. Numerous studies emphasize the relationship between the production of proinflammatory cytokine IL-1β and release factors associated with glutamate toxicity, such as NO-synthase and arachidonic acid [11,12,13,14,15]. In addition, proinflammatory cytokines directly affect synaptic neurotransmission via GABA and glutamatergic receptors [16,17]. Recently, we demonstrated a significant decrease in intracellular pH during neuroinflammation [18]. Such changes may also reduce the efficiency of synaptic transmission and synaptic plasticity, thus resulting in impairments in cognitive functions.

*N*-docosahexanoylethanolamine (DHEA, synaptamide) is an endogenous metabolite and a structural analog of anandamide (*N*-acylethanolamine of arachidonic acid) [19]. However, despite its structural similarity with anandamide, this docosahexaenoic acid (DHA) metabolite does not possess endocannabinoid activity, primarily due to weak binding to cannabinoid receptors (CB), although at the same time it exhibits biological activity that accelerates neurogenesis and synaptogenesis [20,21,22,23,24]. It is because of the pronounced synaptogenic activity of *N*-docosahexanoylethanolamine that the term “synaptamide” was coined [25]. It is known that synaptamide is synthesized in the brain from DHA. At the same time, the synaptamide content in the brain decreases when the DHA intake from food is limited [19] and increases with an increase in the DHA content in the diet [26]. In this regard, it can be assumed that the known beneficial effect of omega-3 polyunsaturated fatty acids on cognitive functions [27] is realized through the activity of the *N*-acylethanolamines of these fatty acids.

Several studies have demonstrated through experiments on cell cultures the beneficial effect of synaptamide on the processes of neurogenesis, neuritogenesis, and synaptogenesis [19,22,28]. The regulation of the neuroinflammation and neurogenesis processes by synaptamide is mainly due to the effect on the cAMP/PKA/CREB signaling pathway. Thus, synaptamide stimulates the production of cAMP on microglia and increases PKA and CREB cAMP-dependent phosphorylation. In addition, a decrease in microglia activity occurs due to the inhibition of NF-κB activation. A c-AMP-dependent CREB-mediated increase in transcriptional activity underlies the stimulation of neurogenesis, neurite growth, and synaptogenesis by synaptamide [21]. In addition, a decrease in proinflammatory cytokine production following synaptamide treatment has been shown in *in vivo* experiments [23]. Synaptamide administration decreased lipopolysaccharide (LPS)-mediated microglial activation and expression of pro-inflammatory cytokines, chemokines, and iNOS in rat brains. In addition, the anti-inflammatory and analgesic activity of synaptamide *in vivo* was shown in a study on a rat model of sciatic nerve chronic constriction injury [29]. DHA has a similar activity [30,31,32]; however, it is assumed that synaptamide has an activity ten times higher than that of DHA [19,21]. At the same time, *N*-acylethanolamines of other fatty acids do not show such activity, which emphasizes the uniqueness and specificity of the biological properties of synaptamide. *In vitro* experiments have shown that synaptamide activity is not blocked by cyclooxygenase and lipooxygenase, but it is reduced by the fatty acid amide hydrolase (FAAH) [33]. This fact also confirms the hypothesis that it is synaptamide that is the main metabolite determining DHA neuroprotective activity. However, the small number of studies devoted to synaptamide’s biological activity dictates the need for further detailed studies of such a promising compound. Our study aims to elucidate the pharmacological activity of synaptamide in LPS-induced neuroinflammation. This model, which mimics neuroinflammation in the development of many neurological diseases, was used to study the effect of synaptamide on the experimental animals’ memory state and the underlying functional, morphological, and biochemical parameters.

## 2. Results

### 2.1. Synaptamide Treatment Prevents Memory Loss

**Figure 1:**
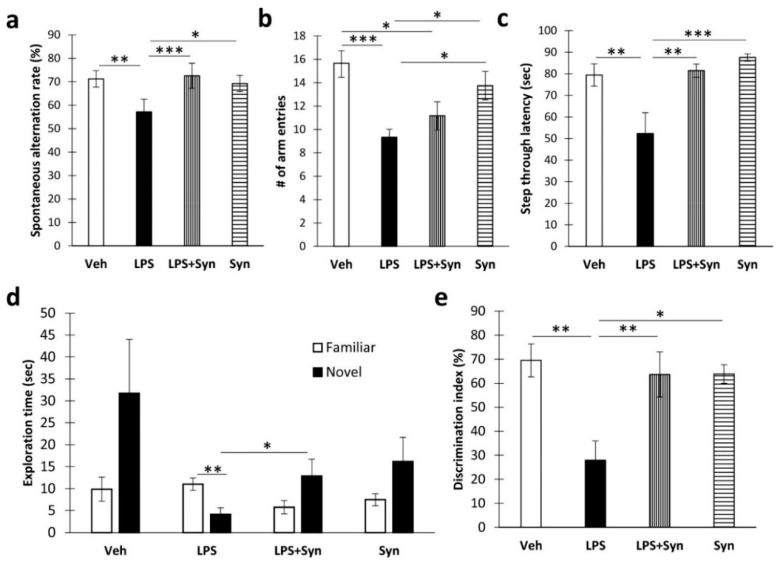
Behavioral effects of lipopolysaccharide (LPS) and synaptamide treatment. (**a**) Spontaneous alternation rate in the Y-maze, %, mean ± SEM, *n* = 15 (number of animals per group). (**b**) Number of arm entries in the Y-maze, %, mean ± SEM, *n* = 15 (number of animals per group). (**c**) Step-through latency in a passive avoidance test, sec, mean ± SEM, *n* = 15 (number of animals per group). Two-way ANOVA with post hoc Tukey test, * *p* < 0.05, ** *p* < 0.01, *** *p* < 0.001. (**d**) Novel object recognition test results: objects’ exploration time, sec. *t*-test, mean ± SEM, *n* = 15 (number of animals per group), ** *p* < 0.01. (**e**) Recognition index, %, mean ± SEM, *n* = 15 (number of animals per group), two-way ANOVA with post hoc Tukey test, * *p* < 0.05, ** *p* < 0.01.

We found that synaptamide treatment prevented the LPS-mediated decrease in spatial working memory during Y-maze testing (LPS: F (3, 60) = 3.99, *p* = 0.049; synaptamide: F (3, 60) = 6.13, *p* = 0.016) (Figure 1a). However, synaptamide treatment did not cause a significant recovery of locomotor activity, which was reduced as a result of neuroinflammation (LPS: F (3, 60) = 19.17, *p* = 0.0001; synaptamide: F (3, 60) = 0.002, *p* = 0.96) (Figure 1b). In addition, synaptamide improved the parameters of long-term memory in animals with neuroinflammation, as shown in the passive avoidance test and the novel object recognition test. For example, in synaptamide-treated animals with neuroinflammation, the step-through latency was significantly higher than in vehicle-treated animals (81.44 ± 3.07 vs. 52.33 ± 9.63, *p* = 0.005). Two-way ANOVA revealed a significant effect of both LPS stimulation and synaptamide treatment on this indicator (LPS: F (3, 60) = 8.88, *p* = 0.004; synaptamide: F (3, 60) = 11.15, *p* = 0.001) (Figure 1c). In a novel object recognition test, the time spent with a novel object in the “LPS” group was significantly lower than the time in the “LPS + Syn” group (4.14 ± 1.48 vs. 12.87 ± 3.83, *p* = 0.047) (Figure 1d). Synaptamide administration prevented a decrease in the recognition index in mice with neuroinflammation (LPS: F (3, 60) = 9.40, *p* = 0.005; synaptamide: F (3, 60) = 4.86, *p* = 0.037) (Figure 1e).

### 2.2. Synaptamide Prevents Synaptic Plasticity Impairment

**Figure 2:**
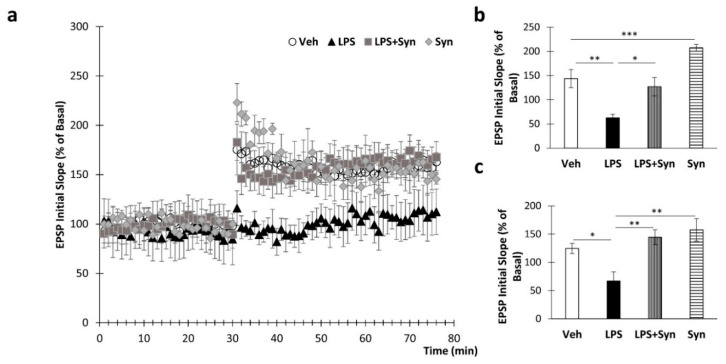
LPS-induced inhibition of long-term potentiation (LTP) is blocked by synaptamide. (**a**) Neuroinflammation blocked tetanus-induced LTP in the Schaffer collateral to CA1 synapses recorded in rat hippocampal slices, but this effect was suppressed by pretreatment of mice with synaptamide. The data are expressed as the mean percentage change in population excitatory postsynaptic potential (EPSP) slope. (**b**) The averaged initial slope measured immediately after LTP, %, *n* = 5 (number of animals per group). Kruskal–Wallis test with post hoc Dunn’s test. (**c**) The averaged initial slope measured at 40 min after LTP, %, mean ± SEM, *n* = 5 (number of animals per group). Kruskal–Wallis test with post hoc Dunn’s test, * *p* < 0.05, ** *p* < 0.01, *** *p* < 0.001.

To study the effects of LPS and synaptamide treatment on synaptic plasticity, long-term potentiation was examined in the CA1 area of mouse hippocampal slices. Stable baseline recordings were obtained for 30 min before tetanus stimulation. Tetanic stimulation of the Schaffer collateral–commissural fibers produced long-term potentiation of field excitatory postsynaptic potentials (EPSPs) in the CA1 area (Figure 2a). The normalized field EPSP slopes in the “LPS” and “LPS + Syn” groups amounted to 95.40% ± 7.94% vs. 157.00% ± 19.03% (*p* = 0.01) of baseline values immediately after tetanus (Figure 2b). A Kruskal–Wallis test revealed a significant overall group difference in this parameter (*p* < 0.001). At 40 min after tetanization, EPSP slopes for “LPS” and “LPS + Syn” were 103.40% ± 17.62% vs. 174.33% ± 20.02% (*p* = 0.009) (Figure 2c). A Kruskal–Wallis test revealed a significant difference in this parameter (*p* = 0.01).

### 2.3. Effect of Synaptamide Treatment on LPS-Induced Dendrite Degeneration

**Figure 3:**
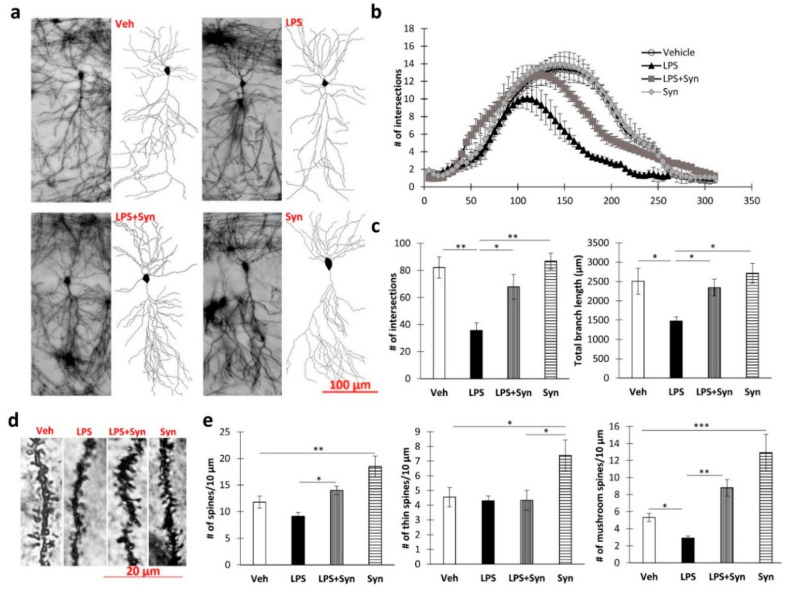
The results of a morphometric study of pyramidal CA1 neurons. (**a**) Representative images of CA1 pyramidal neurons after LPS and synaptamide treatment. (**b**) Number of intersections along the apical dendritic trees at all distances from the soma in CA1 pyramidal neurons after LPS and synaptamide treatment, mean ± SEM, *t*-test, *n* = 5 (number of animals per group). (**c**) The total number of intersections (left), and the total length of apical dendrites (right), mean ± SEM, *n* = 5 (number of animals per group), Kruskal–Wallis test with post hoc Dunn’s test, * *p* < 0.05, ** *p* < 0.01. (**d**) Representative images of CA1 pyramidal neurons’ dendritic spines. (**e**) Density of CA1 pyramidal neurons’ dendritic spines: total—left, thin—middle, mushroom—right, mean ± SEM, *n* = 5 (number of animals per group), Kruskal–Wallis test with post hoc Dunn’s test, * *p* < 0.05, ** *p* < 0.01, *** *p* < 0.001.

Sholl analysis revealed a decrease in CA1 pyramidal neuron complexity when exposed to LPS (Figure 3a). Thus, the degree of dendrite branching at a distance of 125–260 µm from the soma was significantly reduced (*p* < 0.05) compared to the “Veh” group. At the same time, in the “LPS + syn” group, the degree of arborization was significantly higher than in the “LPS” group, at a distance of 130–255 µm from the soma (*p* < 0.05). Synaptamide administration in vehicle-treated mice did not significantly affect the degree of arborization (Figure 3b). A Kruskal–Wallis test revealed a significant difference among the groups in the mean number of intersections in the CA1 pyramidal neurons’ dendritic tree (*p* = 0.015). The subsequent Dunn’s test showed that synaptamide administration prevented an LPS-mediated decrease in mean intersection number: 35.66 ± 5.52 in “LPS” vs. 67.83 ± 9.22 in “LPS + Syn”, *p* < 0.05 (Figure 3c). Synaptamide administration prevented the LPS-induced decrease in dendrite length: 1474.32 ± 110.63 in “LPS” vs. 2345.43 ± 215.91 in “LPS + Syn”, *p* < 0.05 (Figure 3c).

A Kruskal–Wallis test showed a significant overall group difference in dendrite spine density (*p* = 0.039). There was a decrease in the density of mushroom dendritic spines upon induction of neuroinflammation, which was blocked by synaptamide treatment: 2.90 ± 0.22 in “LPS” vs. 8.799 ± 0.97 in “LPS + Syn”, *p* < 0.001. Synaptamide treatment not only prevented the LPS-mediated decrease in the number of mushroom spines but also increased the density of both thin and mushroom spines compared to control (Veh: 4.55 ± 0.65 vs. Syn: 7.38 ± 1.04, *p* = 0.038—thin spines; Veh: 5.30 ± 0.48 vs. Syn: 12.93 ± 2.17, *p* = 0.005—mushroom spines) (Figure 3e). Representative images of dendrites are shown in Figure 3d.

**Figure 4:**
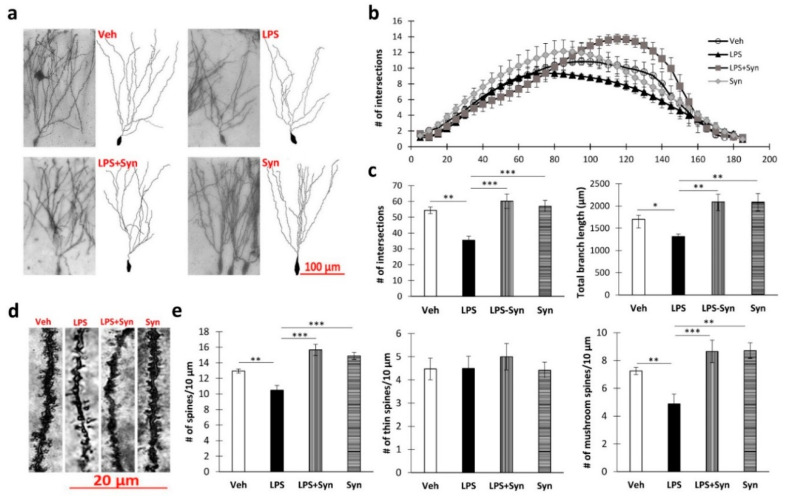
The results of a morphometric study of granular dentate gyrus (DG) neurons. (**a**) Representative images of DG granular neurons after LPS and synaptamide treatment. (**b**) Number of intersections along the dendritic trees at all distances from the soma in DG granular neurons after LPS and synaptamide (number of animals per group). (**c**) The total number of intersections (left), and the total length of apical dendrites (right), mean ± SEM, *n* = 5 (number of animals per group), Kruskal–Wallis test with post hoc Dunn’s test, * *p* < 0.05, ** *p* < 0.01. (**d**) Representative images of DG granular neurons’ dendritic spines. (**e**) Density of DG granular neurons’ dendritic spines: total—left, thin—middle, mushroom—right), mean ± SEM, *n* = 5 (number of animals per group), Kruskal–Wallis test with post hoc Dunn’s test, * *p* < 0.05, ** *p* < 0.01, *** *p* < 0.001.

Sholl analysis of the dentate gyrus granular neurons (Figure 4a) showed that LPS-induced neuroinflammation reduces the number of dendritic intersections at a distance of 90–115 µm from the soma (*p* < 0.05) compared to vehicle-treated animals. At the same time, synaptamide treatment completely prevents such a decrease and contributes to an increase in the number of branches at a distance of 110–145 µm from the soma (Figure 4b). A Kruskal–Wallis test revealed statistically significant differences among the groups in the mean number of intersections (*p* = 0.02) and in the total branch length (*p* = 0.005). Post hoc analysis revealed a significant difference between water-control (“Veh” group) and synaptamide treatment in the mean number of intersections (35.5 ± 2.64—“LPS” vs. 60.2 ± 4.54—“LPS + Syn”, *p* < 0.001) and the total branch length (1314.91 ± 57.27—“LPS” vs. 2091.13 ± 174.77—“LPS + Syn”, *p* < 0.01) (Figure 4c).

A Kruskal–Wallis test revealed a significant overall group difference in dendrite spine density (*p* < 0.001). There was also a decrease in the dendritic spine density in neuroinflammation, which was blocked by synaptamide treatment: 10.47 ± 0.62 in “LPS” vs. 15.66 ± 0.72 in “LPS + Syn”, *p* < 0.001. At the same time, a decrease in the number of spines during neuroinflammation was observed due to mushroom spines, whereas the density of thin spines remained unchanged. Synaptamide treatment not only prevented the LPS-mediated decrease in the number of mushroom spines but also increased their density compared to the control (Veh: 7.24 ± 0.25 vs. synaptamide: 8.71 ± 0.55, *p* = 0.005—mushroom spines) (Figure 4e). Representative images of dendrites are shown in Figure 4d.

### 2.4. Effect of Neuroinflammation and Synaptamide Treatment on Hippocampal Protein Expression

In order to elucidate the mechanisms of synaptamide’s beneficial effects on neuronal morphology and synaptic spasticity in neuroinflammation, we quantified some proteins involved in hippocampal plasticity and neuroinflammatory processes.

**Figure 5:**
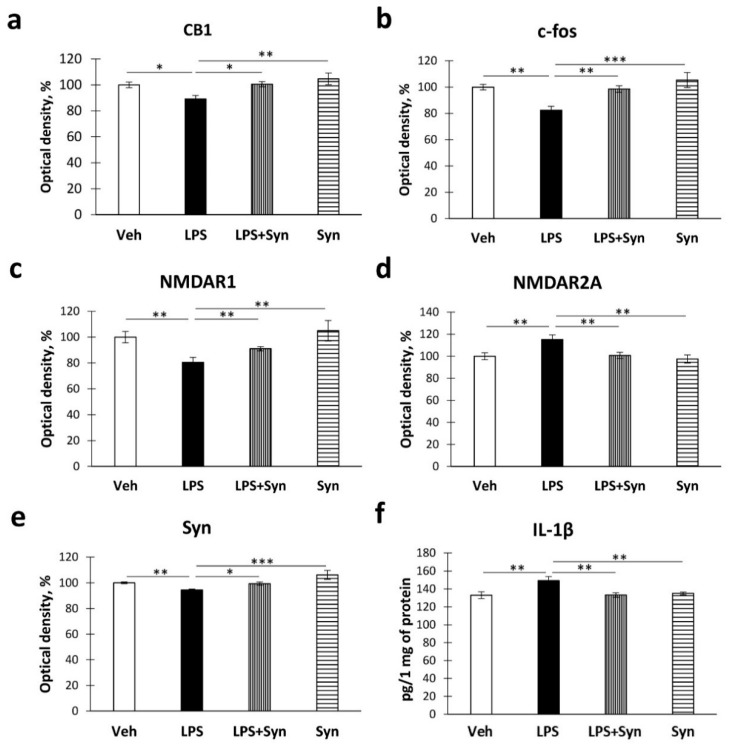
The results of protein quantification by enzyme-linked immunosorbent assay (ELISA). (**a**) Quantification of CB1 expression, %. (**b**) Quantification of c-Fos expression, %. (**c**) Quantification of NMDAR1 expression, %. (**d**) Quantification of NMDAR2A expression, %. (**e**) Quantification of synaptophysin expression, %. (**f**) Quantification of IL-1β expression by ELISA, pg/1 mg of protein. Mean ± SEM, *n* = 10 (number of measurements per group). Two-way ANOVA with post hoc Tukey test, * *p* < 0.05, ** *p* < 0.01, *** *p* < 0.001.

Growing evidence suggests that the endocannabinoid signaling system plays an integral part in the regulation of neuroinflammation processes. Microglial cells actively express CB1 receptors that regulate the release of proinflammatory factors [34]. Using ELISA, we detected a decrease in the level of CB1 receptors in the “LPS” group compared to the “Veh” group (100%, 2.21%—Veh vs. 89.15%, 2.75%—LPS, *p* < 0.05). Synaptamide treatment prevented LPS-mediated decreases in CB1 receptor density. Two-way ANOVA revealed the significance of the LPS stimulation and synaptamide treatment effects on this indicator (LPS: F (3,40) = 7.43, *p* = 0.011; synaptamide: F (3, 40) = 8.58, *p* = 0.007) (Figure 5a).

The proteins encoded by immediate early genes (IEGs), namely Arc and c-Fos, are involved in synaptic plasticity and long-term memory formation [35]. In our study, neuroinflammation significantly decreased the level of c-fos in the hippocampus compared to “Veh” group (100%, 2.10%—Veh vs. 82.43%, 2.92%—LPS, *p* < 0.01). Synaptamide treatment prevented a decrease in the level of c-fos in the hippocampus (LPS: F (3, 40) = 14.21, *p* = 0.001; synaptamide: F (3, 40) = 11.05, *p* = 0.002) (Figure 5b). Given the importance of glutamatergic neurotransmission in the maintenance of synaptic plasticity, in this study we determined the expression of NMDA receptors within the hippocampus. Synaptamide administration reverses the LPS-mediated decrease in NMDAR1 levels (80.53%, 3.80%—“LPS” vs. 91.02%, 1.66%—“LPS + Syn”, *p* < 0.05). Two-way ANOVA revealed the effects of both factors on the NMDAR1 level (LPS: F (3, 40) = 21.58, *p* = 0.0001; synaptamide: F (3, 40) = 4.61, *p* = 0.039) (Figure 5c). At the same time, we observed the opposite situation, with changes in the level of NMDAR2a—namely, synaptamide administration prevents the LPS-mediated increase in the NMDAR2a level (115.10%, 4.21%—Veh vs. 100.72%, 2.84%—LPS, *p* < 0.05). Two-way ANOVA revealed the effect of both neuroinflammation and treatment on the NMDAR2a level (LPS: F (3, 40) = 6.94, *p* = 0.013; synaptamide: F (3, 40) = 5.89, *p* = 0.021) (Figure 5d).

To elucidate the mechanisms of synaptamide influence on LPS-mediated synaptic plasticity impairment, we determined the production of synaptophysin (syn), a protein of presynaptic vesicles, using ELISA. A study of hippocampal synaptophysin production showed that synaptamide treatment prevents an increase in hippocampal syn concentration after LPS administration. Two-way ANOVA showed a significant main effect of synaptamide treatment and LPS stimulation on syn production (LPS: F (3, 40) = 23.04; *p* < 0.0001; synaptamide: F (3, 40) = 18.70, *p* < 0.0001) (Figure 5e).

Assuming the participation of proinflammatory microglia activation in the observed phenomena, at the next stage, we investigated the production of proinflammatory cytokine IL-1β by ELISA and the activity of Iba-1+ microglia immunohistochemically. A study of the proinflammatory cytokine IL-1β production showed that synaptamide treatment prevents an increase in IL-1β hippocampal concentration after LPS administration. Two-way ANOVA showed a significant main effect of synaptamide treatment and LPS stimulation on IL-1β production (LPS: F (3, 40) = 5.08; *p* = 0.028; synaptamide: F (3, 40) = 4.82, *p* = 0.032) (Figure 5f).

**Figure 6:**
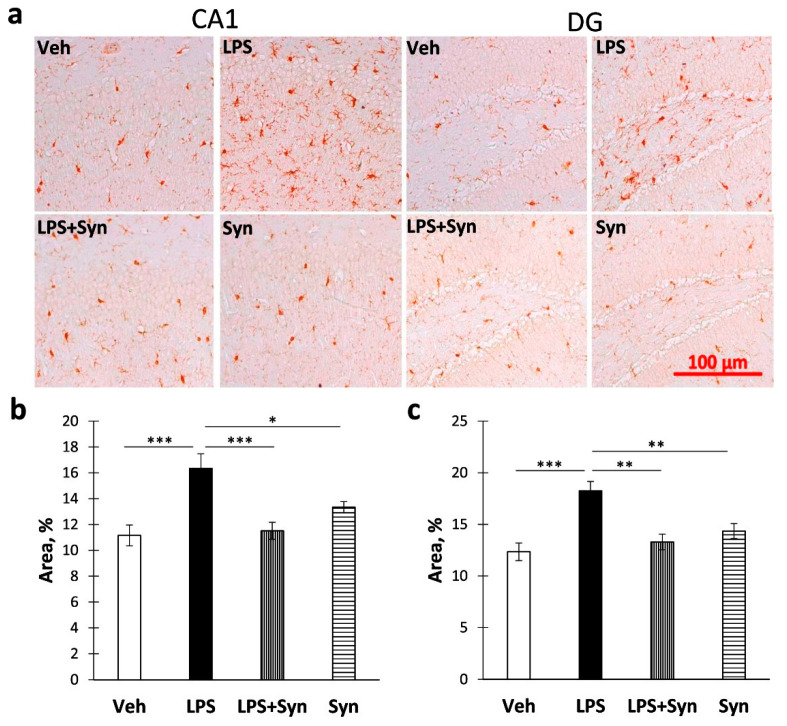
The results of immunohistochemical quantification of Iba1-positive area in CA1 and DG hippocampal areas. (**a**) Representative images of Iba1-positive immunostaining in CA1 and DG hippocampal areas. Scale bar—100 µm. (**b**) Histogram demonstrating the percentage of area covered by Iba1-positive staining in CA1 area. (**c**) Histogram demonstrating the percentage of area covered by Iba1-positive staining in DG. Mean ± SEM, *n* = 5 (number of animals per group). Kruskal–Wallis test with post hoc Dunn’s test, * *p* < 0.05, ** *p* < 0.01, *** *p* < 0.001.

The immunohistochemical study of microglia activity (Figure 6a) showed an increase in Iba-1 immunostaining in CA1 (Figure 6b) area and DG (Figure 6c) within the hippocampus after LPS administration compared to Veh (CA1: 11.16 ± 0.81—“Veh” vs. 16.35 ± 1.13—“LPS”, *p* < 0.001; DG: 12.35 ± 0.85—“Veh” vs. 18.24 ± 0.92—“LPS”, *p* < 0.001). A Kruskal–Wallis test revealed a significant difference in Iba-1 immunoreactivity within the CA1 area (*p* = 0.003) (*p* < 0.001) (Figure 6b) and the DG (*p* = 0.008) across all four groups (Figure 6c).

**Figure 7:**
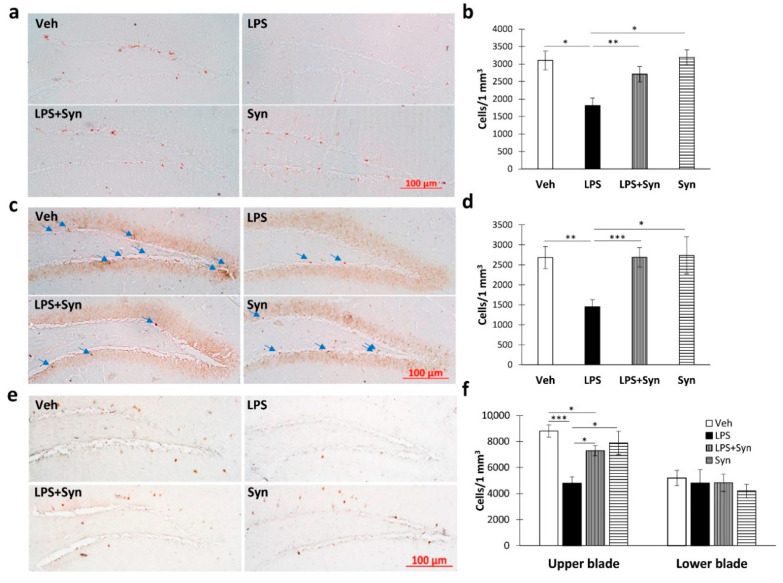
The results of immunohistochemical quantification of protein production in the hippocampal DG subgranular zone (SGZ). (**a**) Representative images of doublecortin-positive cells in the DG SGZ. Scale bar—100 µm. (**b**) Number of doublecortin-positive cells in the DG SGZ. (**c**) Representative images of PCNA-positive cells in DG SGZ. Scale bar—100 µm. Arrows indicate PCNA-positive cells. (**d**) Number of PCNA-positive cells in the DG SGZ. (**e**) Representative images of Arc-positive cells in DG SGZ. Scale bar—100 µm. (**f**) Number of Arc-positive cells in the DG SGZ. Mean ± SEM, *n* = 5 (number of animals per group). Kruskal–Wallis test with post hoc Dunn’s test, * *p* < 0.05, ** *p* < 0.01, *** *p* < 0.001.

Immunohistochemical detection of doublecortin (DCX, a marker of newly formed neurons) showed a decrease in the number of DCX-positive cells in the DG subgranular zone (SGZ) of LPS-treated mice compared to the “Veh” group (3103.43 ± 265.57—“Veh” vs. 1816.89 ± 209.58—“LPS”). Significant differences were found between “LPS” and “LPS + Syn” groups (*p* < 0.01) (Figure 7a). A Kruskal–Wallis test revealed a significant overall group difference in the number of DCX-positive cells within the DG SGZ (*p* = 0.001) (Figure 7b). A similar pattern was observed for PCNA production (a marker of proliferation and reparation) (Figure 7c). We found a decrease in the number of PCNA-positive cells in the DG SGZ of LPS-treated mice compared to the “Veh” group (2682.33 ± 275.61—“Veh” vs. 1451.98 ± 179.28—“LPS”). Significant differences were found between “LPS” and “LPS + Syn” groups (*p* < 0.001) (Figure 7d). A Kruskal–Wallis test revealed a significant overall group difference in the number of PCNA-positive cells within the DG SGZ (*p* = 0.001).

Immunohistochemical study of Arc protein production in the DG SGZ (Figure 7e) revealed its decrease in neuroinflammation, whereas synaptamide inhibits this effect (Figure 7f). A Kruskal–Wallis test revealed a significant overall group difference in the number of Arc-positive cells (*p* < 0.001).

## 3. Discussion

This study demonstrated the beneficial effects of *N*-Docosahexanoylethanolamine (synaptamide) on various behavioral, morphological, and biochemical parameters in mice with LPS-induced neuroinflammation. Studies show that the essential omega-3 polyunsaturated fatty acid DHA has anti-inflammatory activity through the formation of metabolites including neuroprotectins and resolvins [32,36]. However, recent studies demonstrate significant anti-inflammatory activity of such DHA derivatives as synaptamide [21,37]. At the same time, it is known that an increase in cAMP in immune cells reduces the production of pro-inflammatory mediators [38]. The fact that synaptamide increases the production of cAMP in nerve cells suggests that DHA implements most of its anti-inflammatory activity through its synaptamide metabolite. This hypothesis was confirmed in a study by Park et al. [23], in which the anti-inflammatory activity of synaptamide was studied in microglial cell cultures and LPS-induced neuroinflammation in mice. In addition, in cultures of macrophages treated with bacterial lipopolysaccharides, the ability of synaptamide to reduce the production of NO and MCP-1 was shown, which also confirms its anti-inflammatory properties [23,25]. At the same time, the content of synaptamide in the brain decreases when the intake of DHA from food is limited [19] and increases with elevated DHA consumption [26,39].

In our study, we found a beneficial effect of synaptamide on several behavioral indicators in mice with LPS-induced neuroinflammation and tried to identify some of the mechanisms underlying cognitive improvement. As a result of testing in the Y-maze, we found a spatial working memory disturbance in mice with LPS-induced neuroinflammation, which is consistent with the data in the literature [40,41]. Furthermore, in our study, LPS-induced neuroinflammation led to a decrease in the recognition index in the novel object recognition task, which indicates impairment of long-term memory and is also consistent with the data in the literature [9]. These changes were accompanied by an increase in the microglia activity within the CA1 region, a decrease in the complexity of the CA1 pyramidal neurons’ dendritic tree, impaired hippocampal synaptic plasticity, and a hippocampal protein level change. Synaptamide administration to the LPS-treated animals reversed the functional, morphological, and biochemical parameters of the hippocampus that were impaired during neuroinflammation and prevented memory impairment. LPS is known to induce cognitive abnormalities by suppressing long-term potentiation within the hippocampus [25]. At the same time, we showed that synaptamide treatment restored the impaired hippocampal long-term potentiation. Synaptic plasticity is affected by a number of factors, including the state of the synapse, Ca2+ concentration, local potential of the dendritic membrane, etc. Given the fact that synaptic plasticity can occur locally in dendrites without the involvement of somatic output, the state of the dendritic tree is extremely important for the information storage processes [42]. Thus, in our study, the normal level of hippocampal synaptic plasticity may be based on the maintenance of the CA1-pyramidal neurons’ dendritic tree structure. It is known that factors such as NO and cytokines produced by microglia upon activation can inhibit growth and cause neurite retraction [43,44]. The major mechanism of neuronal degeneration inhibition by synaptamide is an increase in cAMP production and CREB phosphorylation [21]. Previous studies have shown that an increase in cAMP production by synaptamide is a consequence of GPR110 receptor stimulation in both CNS microglia and peripheral macrophages. Increased GPR110/cAMP signaling inhibits NF-κB transcription, which leads to a decrease in microglial activity and reduced production of pro-inflammatory cytokines. The maintenance of the presynaptic protein synaptophysin level, which was significantly reduced upon LPS exposure, may be involved in the maintenance of synaptic plasticity during synaptamide treatment. Moreover, previous studies have shown that the decrease in the synaptophysin level following LPS treatment is a microglia-dependent process [45,46]. Thus, synaptamide-induced inhibition of microglial activity prevents a decrease in synaptophysin levels, thus preventing presynaptic disruption and impairment of synaptic plasticity. A decrease in the pro-inflammatory cytokine IL-1β concentration, which can directly reduce the level of synaptophysin, also plays a role in this process [47].

Given the importance of glutamatergic neurotransmission in the maintenance of synaptic plasticity, in this study, we determined the NMDA receptors’ expression within the hippocampus. As is well known, glutamate, being the main excitatory neurotransmitter, plays an important role in the functioning of the nervous system. As previously shown, in LPS-induced inflammation, synaptic plasticity is impaired by suppression of glutamatergic transmission [47,48]. A special role in these processes is played by the release of the pro-inflammatory cytokines IL-1β [49] and IL-6 [50,51]. Suppression of NMDA-dependent LTP may be due to the decrease in the activity of NMDA receptors. In this study, using ELISA, we revealed the NMDAR1 density decreases within the hippocampus following the LPS exposure. This finding is consistent with previous studies in which chronic neuroinflammation reduced the number of NMDAR1-immunopositive cells in the hippocampus [52]. The change in the glutamate receptors’ density may be based on the phenomenon of excitotoxicity, which is often observed in neuroinflammation. This phenomenon is based on the stimulation of glutamate release by pro-inflammatory factors, which ultimately leads to hyperstimulation of glutamate receptors and the entry of toxic Ca^2+^ concentrations into cells [53]. However, different subtypes of NMDA receptors have different properties and functional activities. Thus, functional NMDA receptors are heterotetramers consisting of two dimers containing R1 and R2 subunits. In this case, the homomers of the R2 subunits do not form functional receptors, whereas the homomers of the R1 subunits form channels activated by glutamate or NMDA and generating a current of very low amplitude [54]. This may explain the multidirectional changes in the NMDAR1 and NMDAR2 density upon exposure to LPS and excess glutamate production. Excessive Ca^2+^ entry into cells leads to the degeneration of NMDA-expressing neurons and ultimately to a decrease in the number of functionally active NMDAR1 [52,55]. Since the main role of NMDAR2 is to modulate the functional activity of NMDAR1, a decrease in the amount of active NMDAR1 probably leads to disruption of glutamatergic transmission and a compensatory increase in NMDAR2 density [54]. At the same time, in our study, neither a decrease in the NMDAR1 level nor an increase in the NMDAR2 level was observed in synaptamide-treated animals, which was probably a consequence of the suppression of the inflammatory process within the central nervous system.

Changes in the functioning of the glutamatergic system may be partially associated with the observed decrease in the level of CB1 receptors within the hippocampus following LPS exposure [56]. Synaptamide neuroprotective activity is possibly CB1-mediated and associated with an increase in the level of CB1 receptors, which control NMDAR hyperactivation and associated neuronal dysfunction. This may be due to the fact that activated NMDARs recruit CB1 receptors, which in turn are involved in the blocking of calcium fluxes, resulting in reduced excitotoxicity [56]. At the same time, an increase in the CB1 receptor concentration may be due to excessive production of endocannabinoids by microglia during neuroinflammation, and adaptation to such changes occurs at the protein level [57].

Long-term potentiation, impaired by neuroinflammation, leads to changes in the expression of genes encoding specific proteins required for LTP maintenance and long-term memory formation [58]. These include proteins encoded by immediate early genes (IEGs), namely Arc and c-Fos, the production of which in the hippocampus, as shown in our study, is significantly reduced upon neuroinflammation induction. The role of these proteins in hippocampus-dependent memory formation has been shown in several studies [59,60,61]. We suggest that the synaptamide-induced inhibition of neuroinflammatory processes, preventing hyperstimulation of glutamate receptors and LTP disruption through the maintenance of NMDAR1 levels, normalizes immediate-early gene expression and reverse long-term memory impairments. As previously shown, Arc can alter LTP by regulating actin polymerization and the formation of new dendritic spines [62]. In our study, Arc accumulation within the hippocampal dentate gyrus (DG) in synaptamide-treated animals probably prevented a decrease in dendritic spine density in the DG granular neurons. This inhibitory effect of synaptamide on the decrease in Arc production can lead to the maintenance of normal LTP levels, dendritic spine density, and dendritic tree morphology. Given the leading role of DG in long-term memory, including long-term hippocampus-dependent context learning and recognition memory [63,64], we can explain the beneficial effect of synaptamide on long-term memory indicators assessed in tests for passive avoidance and novel object recognition. For example, in our study, synaptamide reversed the LPS-associated decrease in PCNA and DCX in the DG subgranular zone (DG SGZ) and prevented a decrease in the discrimination index in the NOR test. We believe that the prevention of neurogenesis disturbances is based on a decrease in the neuroinflammation and microglial activation in the DG, as shown in our study. It is known that the production of proinflammatory cytokines is associated with a decrease in neurogenesis [65], whereas the production of anti-inflammatory factors usually stimulates the proliferation of neurons in the DG SGZ [66,67]. Immediate early gene accumulation within the hippocampus is associated with an increase in the dendritic spines’ density on pyramidal neurons [68]. A similar situation was observed in our study, since treatment with synaptamide prevented both a decrease in Arc production and the mushroom dendritic spines’ density on the dendrites of granular neurons.

## 4. Materials and Methods

### 4.1. Animals and Treatments

Male *C57BL/6* mice (3 months old) were used for the experiments. The mice were obtained from the National Scientific Center of Marine Biology, Far Eastern Branch of the Russian Academy of Sciences, Vladivostok, Russia. The animals were housed 3–4 per cage with a 12-h light/dark cycle and ad lib access to chow and water. The temperature (23 °C ± 2 °C) and humidity (55% ± 15%) were constant. All experimental procedures were approved by the Animal Ethics Committee at the National Scientific Center of Marine Biology, Far Eastern Branch, Russian Academy of Sciences (No. 2/2020, 03.02.2020) according to the Laboratory Animal Welfare guidelines and the European Communities Council Directive 2010/63/EU.

Neuroinflammation was induced by intraperitoneal (i.p.) injections of bacterial lipopolysaccharides (LPS, *Escherichia coli* O111:B4, Sigma-Aldrich, St. Louis, MO, USA). The mice (*n* = 80) were divided into the following treatment groups: “Veh” (*n* = 20)—i.p. saline and water by subcutaneous (s.q.) injection on the back; “LPS” (*n* = 20)—i.p. LPS and water by s.q. injection on the back; “LPS + Syn” (*n* = 20)—i.p. LPS and synaptamide by s.q. injection on the back; “Syn” (*n* = 20)—i.p. saline and synaptamide by s.q. injection. The i.p. saline or LPS (750 mg/kg) injections were administered for seven consecutive days. The volume of the i.p.-injected substance was 100 μL. Synaptamide was injected subcutaneously as an aqueous emulsion at a dose of 10 mg/kg daily for seven consecutive days. The state of the emulsion was achieved by continuous shaking with a Multi-Vortex shaker (V-32, Biosan, Latvia). The volume of the substance injected subcutaneously was 100 μL.

### 4.2. N-Docosahexaenoylethanolamine Preparation

N-docosahexanoylethanolamine was obtained from by-products of salmon caught in the Bering Sea. The concentrate of polyunsaturated fatty acid was obtained according to the method of Ermolenko et al. [69]. To obtain ethanolamines, a polyunsaturated fatty acid (PUFA) concentrate was converted into ethyl esters and then treated with ethanolamine. HPLC of PUFA ethanolamides was performed using a Shimadzu LC-8A chromatograph (Shimadzu, Kyoto, Japan) with UV/VIS SPD-20A (205 nm). Separation was carried out using a Supelco Discovery HS C-18 preparative reverse phase column (Sigma-Aldrich, Bellefonte, PA, USA); particle size 10 μm, inner diameter 250 mm × 50 mm. Isocratic elution with ethanol/water (70:30, *v*/*v*) was used. The elution rate was 50 mL/min. Fractions containing synaptamide were collected, evaporated in vacuo and analyzed by gas chromatography (GC) and gas chromatography-mass spectrometry (GC-MS). Synaptamide was a light-yellow oily liquid with a mild odor at room temperature. The purity was 99.4%.

Determination of the fatty acid ethanolamide composition as trimethylsilyl derivatives (TMS-NAE) was performed using gas chromatography (GC) and gas chromatography-mass spectrometry (GC-MS) methods. To obtain TMS-NAE, 50 μL of N, O-bis (trimethylsilyl) trifluoroacetamide (BSTFA) was added to 1 mg of fatty acid ethanolamides and heated to 60 °C for 1 h under argon. Then 1 mL of hexane was added and 1 μL of each silylated fraction was injected into the GC system. To determine the composition of TMS-NAE, a Shimadzu GC-2010 plus chromatograph (Shimadzu, Kyoto, Japan) with a Supelco SLB ™-5 ms capillary column (30 m × 0.25 mm inner diameter) was used (Sigma-Aldrich, Bellefonte, PA, USA), along with a flame ionization detector (Shimadzu, Kyoto, Japan). The components of the mixture were separated under certain conditions: (1) an initial temperature of 180 °C; (2) a heating rate from 2 °C/min to 260 °C; (3) the temperature was maintained for 35 min. The injector and detector temperatures were the same and amounted to 260 °C. GC-MS was used to identify the TMS-NAE structures. Electronic impact spectra were recorded on a Shimadzu TQ-8040 instrument (Shimadzu, Kyoto, Japan) with a Supelco SLB ™-5 ms column (Sigma-Aldrich, Bellefonte, PA, USA) at 70 eV. The same temperature conditions were used as for gas chromatography.

### 4.3. Working Memory

Working memory in mice was assessed using the spontaneous alternation test in the Y-maze. The Y-maze was made of opaque acrylic glass and was composed of 3 equal arms (length: 30 cm; height: 40 cm; width: 10 cm). Each mouse was placed in the center of the maze and was allowed to move freely and explore the surrounding space for 5 min. An arm entry was counted when the animal entered the arm with all four paws. To determine the rate of spontaneous alternations, the total number of entries (N) and the number of “correct” triplets (M, consecutive choices of each of the 3 branches without repeated entries) were counted. The spontaneous alternation rate was calculated using the following formula: R (%) = M × 100/(N − 2).

In addition to working memory, the animal’s locomotor activity was assessed using the Y-maze test. To this end, the total number of entries into the maze arms were counted for each mouse.

### 4.4. Passive Avoidance Test

We used the passive avoidance test to assess the effects of neuroinflammation and synaptamide treatment on the long-term memory of experimental animals [70]. The test device consisted of light and dark chambers separated by a sliding door. During the training phase, the mice were placed into the light chamber and allowed to move freely for 60 s before opening the sliding door. When the animal entered the dark chamber, the door was closed, and after 2 s an electric foot-shock was produced (0.25 mA, 2 s). The test phase was performed 24 h after training, but the electric foot-shock was not applied. To assess long-term memory, we measured the delay time before the mice entered the dark chamber (step-through latency).

### 4.5. Novel Object Recognition

The novel object recognition test was performed according to the method described by Bevins and Besheer [71]. During the training session, each mouse was placed in the middle of the arena with 2 identical plastic objects located on the left and right sides for 10 min. The animal was then returned to the home cage for a 24 h retention interval. For the testing session, each mouse was placed in the middle of the arena, where one of the objects was replaced with a new one. The animal was left in the chamber, where it could freely explore objects for 5 min. The behavior was continuously recorded using a video camera which was placed over the apparatus. The criterion of the exploration of the object was the location of the nose of the animals at a distance not > 2 cm from the object. The discrimination ratio was calculated as the time spent on a new object divided by the total time spent exploring both objects. The objects and arenas were carefully cleaned with 70% ethanol after each animal.

### 4.6. Electrophysiological Recording

After the behavioral test, mice were deeply anesthetized using isoflurane (Laboratories Karizoo, S.A., Barcelona, Spain), decapitated, and brains were removed and transferred to ice-cold artificial cerebrospinal fluid (aCSF) composed of 119 mM NaCl, 2.5 mM KCl, 2 mM MgCl_2_, 0.25 mM CaCl_2_, 26 mM NaHCO_3_, 1 mM NaH_2_PO_4_, and 10 mM d-glucose, pH 7.4, oxygenated with carbogen 95% O_2_, 5% CO_2_. The hippocampus was removed and parasagittal sections with a thickness of 350 μm were prepared using a vibratome (PELCO easiSlicer™, Ted Pella, Inc., Redding, CA, USA). The slices were recovered for 1 h at 33 °C. All recordings were performed in a submersion recording chamber perfused with aCSF (30 °C ± 0.5 °C, 2 mL/min). After placing the slices into the recording chamber, the slices were held in place with a nylon mesh during aCSF perfusion. Hippocampal slices were visualized using an upright microscope (Olympus BX50, Olympus, Tokyo, Japan). The recording extracellular electrode with an outer diameter of 1.5 mm and 10 cm in length was made of borosilicate glass (World Precision Instruments, Sarasota, FL, USA). The monopolar stimulating electrode was made of Pl-Ir Teflon wire (75 µm in diameter, including Teflon coating). The stimuli were triggered using National Instruments Labview (National Instruments Corporation, Austin, TX, USA) 2019 software (10 μs duration, Master8, Microprobes for Life Science, Gaithersburg, MD, USA) using an isolating stimulator (Constant Current Stimulus Isolator, World Precision Instruments, Sarasota, FL, USA). The recordings were made using an intracellular amplifier in bridge circuit mode (Axoclamp 2B, Axon Instruments, Molecular Devices, LLC, Sunnyvale, CA), with a sampling rate of 15 Hz, digitized (PCI 6154, National Instruments Corporation, Austin, TX, USA), analyzed, and filtered using National Instruments Labview 2019 software (National Instruments Corporation, Austin, TX, USA).

The stimulating electrode was placed into the Schaffer collateral fiber tract between the CA2 and CA1 regions. For extracellular recording of field potentials, a recording electrode was placed in the CA1 region in the area of the pyramidal cell dendrites at a distance of no more than 1500 μm, but not less than 300 μm from the stimulating electrode in order to avoid direct stimulation of cells located near the recording sites. A slice was considered suitable for recording if an extra-synaptic potential was observed during stimulation of 0.5 mA, provided that the classic graph of input/output stimulation currents (IO) was obtained. To stabilize the responses, stimulation with a frequency of 1 Hz, 0.4 mA for 30 min was used. For the development of long-term post-tetanic potentiation, the amplitude of the testing stimulus was 70% of the maximum extrasynaptic potential amplitude. LTP was obtained using 100 Hz stimulation for 1 s.

### 4.7. Golgi–Cox Staining

The mice were deeply anesthetized with isoflurane (Laboratories Karizoo, S.A., Barcelona, Spain) using a rodent anesthesia vaporizer (VetFlo ™, Kent Scientific Corporation, Torrington, CT, USA), decapitated, and brains were quickly removed from the skull, rinsed with 0.1 M PBS (+4 °C) and cut into 2 hemispheres. The material was stained with the FD Rapid GolgiStain™ kit (FD NeuroTechnologies, Ellicott City, MD, USA) according to manufacturer’s instructions (http://www.fdneurotech.com/item/0/41/0/733/FD_Rapid_GolgiStain_Kit_large). Cryomicrotome (HM 550, Thermo Scientific, Waltham, MA, USA) was used to cut 100-μm-thick slices. Slices were mounted on gelatin-coated microscope slides, stained, dehydrated, and covered with VectaMount™ Mounting Medium (H-5000, Vector laboratories, San Francisco, CA, USA).

### 4.8. Sholl Analysis

Sholl analysis [72] was performed to evaluate the effect of neuroinflammation and synaptamide treatment on the hippocampal dendrite morphology. ImageJ software (NIH, Bethesda, MD, USA) was used for all image processing and morphological analyses. For dendrite tracing, images for each individual neuron were converted to 8-bit color images. Dendrites were traced using the NeuronJ plugin (http://www.imagescience.org/meijering/software/neuronj/) as previously described [73]. Sholl analysis was performed on the NeuronJ tracings using the Sholl Analysis plugin (http://fiji.sc/Sholl_Analysis). For the Sholl analysis, single animals were selected as the unit of analysis (5 animals per group). For each animal, 2–3 well-stained neurons from CA1 and DG areas were taken for evaluation. When calculating the density of dendritic spines, one mouse (5 per group) was used as the unit of analysis. For each mouse the evaluation was performed on 3 neurons.

### 4.9. ELISA

To determine the CB1, c-Fos, NMDAR1, NMDAR2a, synaptophysin, and IL-1β protein concentration within the hippocampus, the enzyme-linked immunosorbent assay (ELISA) was used. The rats were anesthetized with isoflurane using rodent anesthesia vaporizer (VetFlo™, Kent Scientific Corporation, Torrington, CT, USA) and the hippocampus was quickly extracted, frozen in liquid nitrogen, and stored at a temperature of −70 °C. The following primary antibodies were used: Anti-Cannabinoid Receptor I antibody (1:200, ab23703), Recombinant Anti-c-Fos antibody (EPR21930-238) (1:1000, ab222699), Recombinant Anti-NMDAR1 antibody (EPR2481(2)) (1:1000, ab109182), Anti-NMDAR2A antibody (EPR2465(2)) (1:1000, ab124913), Anti-Synaptophysin antibody (1:1000, ab14692), all of Abcam (Cambridge, UK). For IL-1β quantification IL-1 beta Mouse ELISA Kit (BMS6002, Thermo-Fisher Scientific, Waltham, MA, USA) was used.

For analysis we used both right and left hippocampi. The samples were homogenized using a homogenization buffer consisting of 100 mM Tris, pH 7.4, 150 mM NaCl, 1 mM EGTA, and 1 mM EDTA, 1% Triton X-100, 0.5% sodium deoxycholate with a of protease inhibitors cocktail (cOmplete™, Sigma-Aldrich, Bellefonte, PA, USA) incubated on ice for 15 min, centrifuged (16,000× *g*, 30 min, +4 °C) and the supernatants were collected. A BCA Protein Assay Kit (Pierce, Rockford, IL, USA) was used for determining protein concentration. The samples were diluted with bicarbonate-carbonate coating buffer (100 mM, 3.03 g Na_2_CO_3_, 6.0 g NaHCO_3_, 1000 mL distilled water, pH 9.6) to obtain a 20 μg/mL concentration. Then, 100 μL of samples were added to each well of a PVC microtiter plate (M4561-40EA, Greiner, Kremsmünster, Austria) and incubated at 4 °C overnight. After this, the coating solution was removed, and the plate was washed three times by filling the wells with 200 µL PBS. To block the remaining protein-binding sites in the coated wells, 5% non-fat dry milk (M7409-1BTL, Sigma-Aldrich, St. Louis, MO, USA) was used (2 h at room temperature). After washing, 100 µL of diluted primary antibody was added to each well. The plate was covered with an adhesive plastic and incubated for 2 h at room temperature. After washing, 100 µL of peroxidase secondary antibody solution (1:500, PI-1000-1, Vector laboratories, San Francisco, CA, USA) was added to each well, and the plate was incubated for 2 h at room temperature. After washing, 50 µL of TMB solution (3,3′,5,5′-tetramethylbenzidine, SK-4400, Vector laboratories, San Francisco, CA, USA) was added to each well, and the plate was incubated for 30 min at room temperature before color appeared. After sufficient color was developed, 50 µL of stop solution (1N hydrochloric acid) was added to the wells. The absorbance was measured in an iMark plate spectrophotometer (Bio-Rad, Hercules, CA, USA) at a wavelength of 450 nm. Each sample was analyzed twice and the results were averaged.

### 4.10. Immunohistochemical Studies

Brains were extracted for immunohistochemical studies on the 7th day after the start of treatment. The mice were deeply anesthetized with isoflurane (Laboratories Karizoo, S.A., Barcelona, Spain) using a rodent anesthesia vaporizer (VetFlo ™, Kent Scientific Corporation, Torrington, CT, USA) equipped with a rodent mask. Mice were transcardially perfused with 5 mL PBS (~4 °C), pH 7.2. Then the brain was rapidly removed from the skull, divided into 2 hemispheres, and placed in 4% paraformaldehyde for 12 h. Both hemispheres were used for immunohistochemical study. Then, the material was washed with PBS (pH 7.2). After paraformaldehyde fixation the tissue samples were embedded in paraffin blocks and sectioned to obtain 10 µm slices, using a Leica rotary microtome RM 2245 (Leica, Wetzlar, Germany). The immunohistochemical method used in the study consisted of the following steps: (1) blocking endogenous peroxidase activity: 0.3% H_2_O_2_ solution for 5 min; (2) blocking non-specific antibody binding: 5% BSA in PBS for 1 h; (3) primary antibodies (4 °C, 24 h); (4) secondary antibodies conjugated to horseradish peroxidase: anti-rabbit, 1:200, PI-1000-1; anti-mouse 1:200, PI-2000-1 (both from Vector laboratories, San Francisco, CA, USA); (5) ImmPACT™ DAB Peroxidase Substrate chromogen (SK-4105, Vector Laboratories, San Francisco, CA, USA); (6) washing with 0.1 M PBS (pH 7.2), dehydration and mounting in VectaMount Permanent Mounting Medium (H-5000, Vector laboratories, San Francisco, CA, USA). The following primary polyclonal rabbit antibodies were used: anti-Iba-1 rabbit polyclonal antibodies (1:500, ab108539, Abcam, Cambridge, UK), anti-doublecortin (anti-DCX) antibody (1:500, ab18723; Abcam, Cambridge, UK), anti-PCNA mouse monoclonal antibodies (1:500, ab29, Abcam, Cambridge, UK), anti-Arc ab183183 rabbit monoclonal antibodies (1:200, ab183183, Abcam, Cambridge, UK).

Images were obtained on a Zeiss Axio Imager microscope equipped with an AxioCam 503 color and AxioVision software (Carl Zeiss, Oberkochen, Germany). The images were processed and analyzed using ImageJ software (NIH, Bethesda, MD, USA). Processing of each micrograph included the following steps: conversion to an 8-bit image; subtracting the background (rolling ball radius = 50); contrast enhancement. To measure the area of marker staining, the necessary area was selected, and the percentage of the colored area was calculated. When calculating DCX-, PCNA- and Arc-immunopositive cells/mm^3^, the following formula was used: d = (10^6^ × n)/(S × l), where n is the number of cells; S is the area of the granule cell area (μm^2^); l is the thickness of the slice, and 106 is the conversion coefficient of μm^2^ to mm^2^. Counting Arc-positive cells was carried out in the granule layer of the DG. DCX- and PCNA-positive cells were counted strictly in the DG subgranular zone (SGZ). The quantification of Iba-1-, DCX-, PCNA-, and Arc-immunopositive cells was performed using every 4th section. All measurements were performed by an operator who was blinded to the identity of the sections. For calculations, 5 sections were used from each animal. For statistical processing, the values obtained for each individual animal were averaged.

### 4.11. Statistical Analysis

Data are presented as the means ± SEM. ‘n’ represents the number of animals. All data were tested for normal distribution using the Shapiro–Wilk test. Since the data obtained by the behavioral tests and ELISA were normally distributed, they were subjected to statistical analysis using two-way ANOVA followed by a post hoc Tukey multiple comparison test. The data obtained by the electrophysiological recording, histology, and immunohistochemistry were subjected to statistical analysis using the Kruskal–Wallis test with the post hoc Dunn’s multiple comparison test. A value of *p* < 0.05 was considered to indicate a statistically significant difference. Sholl analysis data were subjected to statistical analysis using a Student’s *t*-test. For all studies, one animal was used as the analysis unit. All statistical tests were performed using Microsoft Excel software (Microsoft, Tulsa, OK, USA) and GraphPad prism 4 (GraphPad Software, San Diego, CA, USA).

## 5. Conclusions

*N*-docosahexanoylethanolamine (synaptamide) administration to animals significantly improved hippocampus-dependent memory, prevented synaptic plasticity impairments, neuronal degeneration, and neurogenesis deterioration. The likely basis of the phenomena described above is the powerful anti-inflammatory activity of synaptamide, as shown in our study and several previous works.

## Abbreviations

Synaptamide	*N*-docosahexanoylethanolamine
LPS	lipopolysaccharides
DG SGZ	dentate gyrus subgranular zone
DCX	doublecortin
PCNA	proliferating cell nuclear antigen
LTP	long-term potentiation
ELISA	enzyme-linked immunosorbent assay
ANOVA	one-way analysis of variance
FAAH	fatty acid amide hydrolase
DHA	docosahexaenoic acid
CB1	cannabinoid-1 Receptor
NMDAR	*N*-methyl-d-aspartate receptor
PBS	phosphate-buffered saline
Veh	vehicle
Syn	Synaptophysin
aCSF	artificial cerebrospinal fluid
